# Patterns of physical and psychological development in future teenage mothers

**DOI:** 10.1093/emph/eot016

**Published:** 2013-08-16

**Authors:** Daniel Nettle, Thomas E. Dickins, David A. Coall, Paul de Mornay Davies

**Affiliations:** ^1^Centre for Behaviour and Evolution, Institute of Neuroscience, Newcastle University, Newcastle, UK; ^2^Department of Psychology, Middlesex University, London, UK; ^3^Community, Culture, and Mental Health Unit, School of Psychiatry and Clinical Neurosciences, University of Western Australia, Crawley, Australia; and ^4^School of Medical Sciences, Edith Cowan University, Joondalup, Australia

**Keywords:** life history theory, development, early reproduction, reproductive strategy

## Abstract

**Background and objectives:** Teenage childbearing may have childhood origins and can be viewed as the outcome of a coherent reproductive strategy associated with early environmental conditions. Life-history theory would predict that where futures are uncertain fitness can be maximized through diverting effort from somatic development into reproduction. Even before the childbearing years, future teenage mothers differ from their peers both physically and psychologically, indicating early calibration to key ecological factors. Cohort data have not been deliberately collected to test life-history hypotheses within Western populations. Nonetheless, existing data sets can be used to pursue relevant patterns using socioeconomic variables as indices of relevant ecologies.

**Methodology:** We examined the physical and psychological development of 599 young women from the National Child Development Study who became mothers before age 20, compared to 599 socioeconomically matched controls.

**Results:** Future young mothers were lighter than controls at birth and shorter at age 7. They had earlier menarche and accelerated breast development, earlier cessation of growth and shorter adult stature. Future young mothers had poorer emotional and behavioural adjustment than controls at age 7 and especially 11, and by age 16, idealized younger ages for marriage and parenthood than did the controls.

**Conclusions and implications:** The developmental patterns we observed are consistent with the idea that early childbearing is a component of an accelerated reproductive strategy that is induced by early-life conditions. We discuss the implications for the kinds of interventions likely to affect the rate of teenage childbearing.

## Abstract (precis)

The developmental patterns of teenage mothers are consistent with the idea that early childbearing is a component of an accelerated reproductive strategy induced by early-life conditions. The implications for interventions likely to affect the rate of teenage childbearing are discussed.

## INTRODUCTION

Most women in Western populations delay the onset of childbearing. However, there is a small minority who become mothers before the age of 20. This ‘teenage childbearing’ phenomenon continues to attract public health interest and policy interventions [1–3], although the basis for considering it a major problem is debatable [4–6]. Policy makers often regard teenage childbearing as a mistake, stemming from lack of skills and knowledge surrounding contraception and sexual relationships [2, 7]. However, the contention that contraceptive behaviour or knowledge is a major causal factor is not well supported by evidence [1, 8, 9]. Moreover, programmes of intervention that provide contraceptive education to adolescents have been found to have no effect on the rate of teenage childbearing [10–12].

Policy makers have viewed this phenomenon as the outcome of ‘poor’ reasoning, and it is assumed that better reasoning will lead to delayed reproduction [13]. An alternative perspective holds that early childbearing is part of a coherent reproductive strategy for some women. Indeed, women’s ideal age for parenthood, surveyed at age 16 in the National Child Development Study (NCDS) (see below), is generally a good predictor of their subsequent actual age at first pregnancy [14]. Such desires could be seen as indicative of peer pressure imposing a social norm within such populations, but stable pro-natal attitudes of this sort also require an explanation, and could easily be symptomatic of a reproductive strategy [13]. In addition, teenage mothers reach menarche relatively early [15], suggesting more rapid maturation.

Reproductive strategies differ between and within species. Life-history theory captures these differences [16]. A key assumption is that organisms will act to maximize their average lifetime inclusive fitness, and that selection will have led to the evolution of proximate mechanisms that enable physiological and behavioural calibration to local ecological contingencies [17]. The degree of calibration will vary across species from fixed to more plastic strategies. Those that inhabit relatively stable ecological niches are more likely to have low levels of plasticity compared with generalists or those from stochastic ecologies [18–20]. Within a species, where different ecologies are populated, we should expect to see different phenotypic responses to maximize inclusive fitness.

Whether or not an organism is high or low on plasticity, their phenotype is regarded as the outcome of selection operating within the parameters of key trade-offs. ‘Trade-offs represent the costs paid in the currency of fitness when a beneficial change in one trait is linked to a detrimental change in another’ [21]. One key trade-off is that between current and future reproduction. Physiologically this amounts to a decision about when to stop investing in somatic capital (growth and maintenance) and divert energy into reproduction [17, 22]. Some species have a total commitment to this decision, including Pacific salmon, whose bodies deteriorate during spawning as they divert all of their somatic capital into reproduction. They die immediately after this event. Other species, including our own, have a mixed allocation across lifespan, and in our case we have a lengthy pre- and post-reproduction life [23].

Within species variation in timing of first reproduction should be sensitive to local ecology. A resource rich ecology will enable a relatively lengthy investment in somatic capital and a consequent delay in reproduction. Where the ecology is stressed, and resource acquisition uncertain, the somatic investment should stop sooner, and reproduction will commence earlier [24]. The trade-off between quality and quantity of offspring will also provide selection pressure. Ecological stress can lead to increased reproduction, effectively as a bet-hedging strategy. Better resources allow for investment in more robust, higher quality offspring [25].

Human populations in the developed world are not uniform in their ecological niche, and do not have equal access to resources. This leads to distinct life-history differences in terms of morbidity and mortality across socioeconomic gradients [26]. There are also differences in reproductive strategy, such that low socioeconomic status neighbourhoods carry a higher risk of teenage pregnancy and motherhood [3, 13, 27–29]. Life-history theory leads us to expect key individual differences in behaviour and physical growth between those who engage in early reproduction compared with those who are relatively delayed. Thus, teenage motherhood can be seen as an extreme end of a niche-specific early fertility strategy. The average age of first birth in poorer neighbourhoods will be lower than that in wealthier boroughs, but not all reproduction will begin during teenage years in deprived areas [30]. For those who do reproduce during their teenage years we must look to additional differences between mothers, and idiosyncratic ecological issues, beyond a general socioeconomic categorization.

Belsky *et al.* [31] proposed that adverse early-life conditions—specifically, low parental investment and family stress—induce accelerated reproductive strategies as an adaptive response. Many studies have observed associations consistent with this hypothesis, such as those between low birthweight and early menarche [32–34], poor parent–child relationships and early menarche [35–38], or between stressful family environment and age at first sexual activity or conception [39, 40]. It is hard to separate out genetic and environmental explanations for these associations, given that there are established heritable effects on pubertal maturation [41], and there could be genetic correlations between these factors and parenting behaviours [42, 43]. However, evidence from genetically informative study designs [36], and experimental animal models [44, 45], suggests that the relationship between early-life inputs and subsequent reproductive strategies may be partly causal. Gene × Environment interactions, whereby people with some genotypes are more responsive than others to the effect of rearing conditions, are also plausible [46].

If teenage childbearing is the outcome of a coherent reproductive strategy, and if that strategy is induced by early environmental conditions, then we can predict that future teenage mothers will differ from their peers in many ways beyond their knowledge about contraception. Moreover, these differences should be evident well before the childbearing years. Physically, we should expect relatively poor growth very early in life, since growth immediately before and after birth is highly sensitive to maternal investment [47, 48]. This should however be coupled with earlier puberty, and because of the relationship between pubertal maturation and stature increase [49], also with earlier cessation of stature growth. Early puberty requires rapid weight gain in middle childhood [50, 51], and thus we might additionally predict this pattern in future young mothers.

At the psychological level, Belsky *et al.* [31] suggested that adverse rearing conditions should be reflected in increased levels of emotional and behavioural problems in childhood, and that these mediate the acceleration of reproductive strategy. Associations have been reported between teenage childbearing and conduct problems in adolescence [52], but there is a paucity of quantitative research examining emotional and behavioural adjustment earlier in childhood in future teenage mothers. The strategic view of teenage childbearing also suggests that future teenage mothers should have a motivational orientation towards early childbearing, and this should be significantly before first conception. Consistent with this view, Maestripieri *et al.* [53] found that adolescent women from father-absent households, who are prone to show accelerated reproductive strategies, show a greater preference for images of infants than their peers.

In this article, we use longitudinal data from the NCDS to compare the developmental profiles of a group of young women who became teenage mothers with those of a control group who did not. We examine physical variables (weight and height, weight and height gain, pubertal development, timing of menarche), and psychological variables (psychological adjustment in childhood, reproductive intentions at adolescence). As outlined earlier, we predict that the future young mothers will be characterized by poorer growth very early in life, rapid weight gain in middle childhood, early menarche and pubertal maturation and the early cessation of growth. Psychologically, we would expect to see negative emotional symptoms and behavioural adjustment problems in childhood, and a motivational orientation to early parenthood that is detectable by adolescence. We also investigate exposure to contraceptive education at age 16, to test for effects of lack of knowledge.

Several of the developmental differences we predict have been found in previous research (e.g. early menarche [14], reduced adult stature [54], unhappiness in childhood [55] and idealization of early parenthood [28] are all associated with teenage childbearing). However, not all studies control rigorously for socio-economic position. This is important, as teenage childbearing is concentrated in the poorest social strata [56], and thus future teenage mothers will differ from the rest of the population in many ways that are related to poverty, but not directly related to their reproductive schedules. In this study, we compare future young mothers only to a socioeconomically matched control group to mitigate this problem, and to identify precursors that are specific to teenage childbearing. Moreover, no previous study has examined all the physical and psychological antecedents in a single investigation. The NCDS has exceptionally rich longitudinal data, including a wide variety of different measures, allowing this order of analysis. We can therefore compare the strength of association across different types of variables to investigate the relative strengths of say, depression in late childhood, early menarche and lack of contraceptive education, as individual predictors of teenage childbearing.

## METHODS

No separate ethical approval was required for this research, as it was based on a secondary analysis of an existing, anonymous data set. Written consent for the storage of data was given by the parents of all cohort members (CMs), and, in adulthood, by the CMs themselves.

### Study population and design

We used data from the NCDS, a longitudinal study of all children born in the UK between 3 March and 9 March 1958. Extensive medical and sociological data were gathered at the time of birth, at 7, 11, 16 and 23 years, using perinatal hospital data, physician examination and interviews with parents, teachers and the CMs themselves. The NCDS is ongoing.

We employed a case control design for the following reasons. First, it is advantageous for studying dynamic populations in which follow-up is difficult. Second, it is effective for examining outcomes with a long latency period between exposure and manifestation—in this study this is up to 20 years. Third, it can be used to examine multiple risk factors for development of the focal variable. Given that longitudinal data have not been collected with our specific hypotheses in mind we recognize that total control is impossible to achieve. To this end we regard this study as an exploratory proof of concept.

Our initial sample included all female CMs whose gestational age was known and was >259 days (term), and who were still in the study at age 23. From these 5152 women, 600 reported having a child before their 20th birthday (the ‘case’ group). Socioeconomic position in 1958 was primarily measured using the Registrar General’s social class framework [57], a five-point scale based on occupational ranking.

To control for family socioeconomic position, we selected a set of controls such that the frequency distribution of the social class of the CM’s mother’s husband (variable *n*492), and the social class of CM’s mother’s father (variable *n*526), was the same in the case and control groups. This included selecting controls with missing values of these variables to correspond to cases with missing values. Selection of controls where there were more than needed who met the criteria was done by lowest NCDS serial number. One case could not be matched due to a unique combination of social class variables and was excluded from the study. Thus, the ‘case’ and ‘control’ groups (*n* = 599) are identical in terms of their distributions of household social class at the time of birth, and social class background of the CM’s mother, although they are unrepresentative of the NCDS women as a whole (see Table 1). The case and control groups do not differ in gestational age (cases: mean 283.31, SD 10.35; controls: mean 283.05, SD 9.70, *t*_1196_ = 0.46, n.s.).

**Table 1.** Frequencies (percentages) of different social classes of mother’s husband, and mother’s father, in the case and control groups, and in women meeting the inclusion criteria from the NCDS cohort as a whole

Class category	Whole cohort	Cases and controls
Mother’s husband		
I	229 (4.4)	3 (0.5)
II	687 (13.3)	35 (5.8)
III	3010 (58.4)	346 (57.8)
IV	601 (11.7)	105 (17.5)
V	409 (7.9)	70 (11.7)
Students	4 (0.1)	0 (0)
Single, dead, away	114 (2.2)	25 (4.2)
Retired	1 (0.01)	0 (0)
Missing data	97 (1.9)	15 (2.5)
Mother’s father		
I	115 (2.2)	3 (0.5)
II	673 (13.1)	47 (7.9)
III	2266 (44.0)	236 (39.4)
IV	633 (12.3)	103 (17.2)
V	586 (11.4)	95 (15.9)
Unemployed, sick	36 (0.7)	3 (0.5)
Dead, away	394 (7.7)	52 (8.7)
Retired	60 (1.2)	6 (1.0)
Missing data	289 (7.6)	54 (9.0)

### Measures

#### Physical development

Our physical development measures include birthweight (oz), weight (kg) and height (m) measured at the ages of 7, 11, 16 and 23. We also used these variables to calculate the gains in weight and height between 7 and 11, 11 and 16 and 16 and 23. Pubertal development was assessed at 11 and 16, with physicians assessing breast development (scales 1–5 at age 11, absent/intermediate/adult at age 16) and pubic hair (scales 1–5 at age 11, absent/sparse/intermediate/adult at age 16). We treat the age 11 pubertal development variables as continuous, and for the age 16 variables, we contrast ‘adult’ (the modal response) with ‘non-adult’ (the other options combined). Age at onset of menses is reported twice in the NCDS data: by the girl being asked during physician examination at age 16, and by mother’s report in an interview at age 16. Once responses of ‘Not yet started’ and ‘Age unknown’ have been deleted from both variables, the two correlate at *r* = 0.72 (*P* < 0.001). Here, we use the mother report as it has more than 100 more complete records for our case group.

#### Psychological development

At ages 7 and 11, CMs’ teachers assessed their behaviour using items from the Bristol Social Adjustment Guides (BSAG) [58]. The teachers indicated whether a large number of classes of behaviour indicating poor adjustment were present (yes = 1/no = 0). These ratings give an overall maladjustment score (BSAG total; higher score indicates worse adjustment), and scores for 12 subscales (unforthcomingness, withdrawal, depression, anxiety about acceptance by adults, hostility towards adults, writing off adults and standards, anxiety about acceptance by children, hostility towards children, restlessness, inconsequential behaviour, miscellaneous symptoms and miscellaneous nervous symptoms). The subscale scores all had a strong mode at zero, and so we have treated them as dichotomous (zero score/non-zero score). The BSAG total scores did not have a mode at zero, but were skewed, and so we have square root transformed them for the purposes of *t*-tests.

At age 16, CMs were asked in an interview to state the ideal age to get married, and the ideal age to start a family. Responses were coded using a series of categories (16 or 17, 18 or 19, 20 or 21, 22–25, 26–30, over 30). We have reconverted these categories into ages using category mid-points (30 for ‘Over 30’), but since the resulting distribution is non-normal, we use non-parametric statistics to test for differences in these variables. In the same interview, CMs were asked whether they had lessons about conception in the context of sex and relationships education at school, and whether they felt that they had been provided with enough information about conception.

### Analysis

As our design controls for socioeconomic position, and the CMs do not differ in age, our statistical analyses are very simple. We compare variables between the case and control groups, reporting odds ratios (ORs) and their confidence intervals (CIs) for dichotomous variables, and *t*-tests or non-parametric Mann–Whitney *U*-tests for continuous ones. We report Cohen’s *d* [59] as a measure of effect size where appropriate. Note that we do not use paired statistics. Since around 150 cases have a father and a maternal grandfather from class III, for example, it would be arbitrary to match each case to one particular control for statistical purposes (and there would be many thousands of equally valid matchings). Instead, our design ensures that the overall socioeconomic profiles of the case and control groups do not differ, but the comparisons are between the group means or frequencies.

## RESULTS

### Growth and physical development

The cases were on average significantly lighter than the controls at birth (Table 2), and tended to be lighter at age 7 (*P* = 0.06). All differences in weight and also in weight gain were non-significant after age 7. The cases were significantly shorter than the controls at 7 and 11, and then again at 23. The height gain 7–11 and 11–16 was no different for cases and controls (data not shown). However, the height gain between 16 and 23 was significantly less for the cases than controls (*t*_788_ = −4.49, *P* < 0.01, *d* = −0.32). The mean height gain 16–23 for the cases was 0.7 cm, compared to 1.5 cm for the controls.

**Table 2.** Comparison of the case and control groups for physical development variables

Measure	NCDS variable	Cases	Controls	Effect size
Birthweight (oz)	*n*574	114.81 (6.93)	116.81 (16.91)	−0.12*
Weight, age 7 (kg)	dvwt07	23.12 (3.46)	23.55 (3.68)	−0.12
Weight, age 11 (kg)	dvwt11	36.73 (7.69)	37.54 (7.52)	−0.11
Weight, age 16 (kg)	dvwt16	54.52 (8.83)	54.19 (8.29)	0.04
Weight, age 23 (kg)	dvwt23	58.16 (10.03)	58.37 (8.96)	−0.02
Height, age 7 (m)	dvht07	1.208 (0.057)	1.220 (0.060)	−0.21*
Height, age 11 (m)	dvht11	1.436 (0.071)	1.447 (0.073)	−0.15*
Height, age 16 (m)	dvht16	1.600 (0.061)	1.607 (0.064)	−0.11
Height, age 23 (m)	dvht23	1.605 (0.065)	1.621 (0.069)	−0.25*
Breast development, age 11	*n*1531	1.98 (0.93)	2.04 (0.95)	−0.06
Pubic hair, age 11	*n*1532	1.86 (0.93)	1.86 (0.89)	0
Breast development, age 16	From *n*2005	Adult 258/non-adult 111	Adult 268/non-adult 155	OR 1.34*
Pubic hair, age 16	From *n*2006	Adult 222/non-adult 133	Adult 244/non-adult 172	OR 1.18
Age at menarche	From *n*2648	12.57 (1.33)	12.86 (1.25)	−0.23*

Given are descriptive statistics for each group (means and standard deviations or frequencies, as appropriate), and effect size of the case–control comparison (Cohen’s *d* or OR, as appropriate). *P < 0.05.

There was no difference in ratings of breast or pubic hair development at age 11 between cases and controls (*t*_946_ = −0.92, n.s.; *t*_945_ = 0.05, n.s.). However, at age 16, cases were more likely to be judged to have adult breasts than the controls (marginally significant: OR = 1.34, 95% CI 1.00–1.81, *P* = 0.05). The odds of being judged to have adult pubic hair were not significantly different between cases and controls (OR = 1.18, 95% CI 0.88–1.57). Menarche was significantly earlier in the cases than controls (*t*_859_ = −3.35, *P* < 0.01, *d* = −0.23; Table 2), with a mean difference of 0.29 years.

### Psychological development

At age 7, the cases had higher total BSAG scores than the controls (*t*_1095_ = 5.77, *P* < 0.01, *d* = 0.35). At age 11, the difference had become more marked (*t*_1034_ = 7.25, *P* < 0.01, *d* = 0.45). Table 3 shows the OR for having a non-zero score on each of the BSAG subscales. At age 7, cases were significantly more likely to have a non-zero score than controls for unforthcomingness, depression, hostility towards adults, writing off adults and standards, inconsequential behaviour, and miscellaneous symptoms. At age 11, cases were significantly more likely to have a non-zero score than controls on all subscales except for withdrawal and anxiety about acceptance by adults. Effect sizes for the BSAG subscales were generally substantial, with a mean OR of 1.82 at age 11 (Table 3).

**Table 3.** OR (95% CIs) for receiving a non-zero score on each of the BSAG subscales, for cases versus controls, at ages 7 and 11

Scale	Age 7	Age 11
Unforthcomingness	1.50* (1.18–1.90)	1.30* (1.02–1.66)
Withdrawal	1.00 (0.72–1.38)	1.34 (0.99–1.83)
Depression	1.64* (1.29–2.09)	2.28* (1.78–2.93)
Anxious accept. adults	1.11 (0.87–1.41)	1.29 (0.99–1.67)
Host. adults	1.95* (1.49–2.56)	2.00* (1.52–2.62)
Writing off adults	1.79* (1.32–2.19)	1.54* (1.20–1.97)
Anxious children	1.11 (0.78–1.72)	1.59* (1.12–2.25)
Host. children	1.22 (0.90–1.72)	2.62* (1.87–3.68)
Restlessness	1.30 (0.94–1.79)	2.43* (1.67–3.34)
Incons. behaviour	1.68* (1.32–1.85)	1.75* (1.37–2.24)
Misc. symptoms	1.45* (1.13–1.85)	1.69* (1.31–2.17)
Misc. nervous	1.12 (0.74–1.70)	1.97* (1.19–3.26)

*P < 0.05.

The case group gave a significantly lower mean ideal age for marriage than the controls (Table 4; Mann–Whitney *U*-test: *z* = 7.77, *P* < 0.01). The case group also had significantly lower mean ideal ages for starting a family than the controls (Mann–Whitney *U*-test: *z* = 7.07, *P* < 0.01). Within the case group, 15.8% reported having had no sex education lessons about conception, compared to 12.8% of the controls (difference not significant: OR 1.28, 95% CI 0.87–1.89). Asked whether they needed more information about conception, 34.3% of the cases answered ‘yes’ or ‘maybe’. This compared to 30.7% of the matched controls (difference not significant: OR 1.12, 95% CI 0.95–1.49).

**Table 4.** Comparison of the case and control groups for psychological development variables

Variable	NCDS variable	Cases	Controls	Effect size
BSAG total score, age 7	*n*455	9.08 (8.29)	6.62 (7.36)	0.35*
BSAG total score, age 11	*n*1008	10.17 (9.53)	6.43 (7.10)	0.45*
Ideal age for marriage	From *n*2809	20.66 (2.54)	21.81 (2.26)	−0.48*
Ideal age for family	From *n*2810	22.67 (2.75)	23.96 (2.55)	−0.49*
No lessons about conception	From *n*2825	Yes 63/no 335	Yes 58/no 396	OR 1.28
Needs more info about conception	From *n*2858	Yes 129/no 247	Yes 135/no 305	OR 1.12

Given are descriptive statistics for each group (means and standard deviations or frequencies, as appropriate), and effect size of the case–control comparison (Cohen’s *d* or OR, as appropriate). *P < 0.05.

## DISCUSSION

Our results indicate that the differences between British women who initiate childbearing early, and their peers who do not, are apparent well before adolescence. Future young mothers in the NCDS cohort were significantly lighter than their peers at birth, and by age 7, lagged behind their peers in terms of height. Between 7 and 16, future young mothers caught up somewhat in terms of height, and particularly in terms of weight, though the difference in weight gain between 7 and 16 was not statistically significant. We note the similarity here to the growth profile of those at risk for cardiovascular and metabolic problems later in life; low weight at birth and in early childhood, followed by relatively rapid weight gain in middle childhood [60]. Thus, accelerated reproductive schedules may have similar developmental origins. Our future young mothers also showed signs of accelerated pubertal maturation, with more adult breast development at 16, and an average age at menarche around 4 months younger than the controls. They also gained very little height after 16 compared to their peers, suggesting early termination of growth and an accelerated transition from adolescence to adulthood. The effect sizes for physical differences between future young mothers and controls were generally small [59], with the difference in timing of menarche providing the largest effect.

The psychological variables reveal increased levels of emotional and behavioural disturbance at age 7 and, more strongly, at age 11. In contrast to the physical differences, the effect sizes for the psychological variables are substantial, with the odds of depression and hostility at age 11, for example, being over twice as high in the future young mothers as in the control group. Previous research has found that conduct disorder, but not affective problems such as depression, in adolescence, is predictive of teenage pregnancy [52]. However, using a psychological assessment in childhood, we found that both conduct problems and affective problems were more prevalent in future young mothers than in controls. In fact, increased emotional and behavioural disturbance in the future young mothers was consistent across all the subscales of the BSAG at age 11. Coupled with this was an idealization of earlier marriage and earlier childbearing by age 16. Thus, the psychological variables suggest a picture of poor adjustment and negative emotionality in mid- to late-childhood, associated with a tendency to reproduce young that is already in place by age 16. This evidence accords with recent qualitative studies, which have suggested that unhappiness in childhood is often a precursor to teenage motherhood, and that it is generally experienced as a positive life development [4, 5, 61].

The pattern of psychological development—unhappiness in childhood alongside a desire for parenthood—neatly mirrors the physical one of poorer childhood growth, but precocious development at and after puberty. Taken together, the physical and psychological trajectories are consistent with the idea of a facultative accelerated reproductive strategy being triggered by adverse early experience [31]. However, we note that with our current data, we can only document the different developmental trajectory of future young mothers; we cannot separate out the possible genetic and environmental influences causing it. There is good evidence for both genetic and environmental influences on, for example, age at menarche [36, 41], and Gene × Environment interactions are also likely to be important.

We should note by way of caution that the case–control comparisons reported here aggregate all the future young mothers together, and all the controls together. Thus, our analyses do not reflect the fact that there may be multiple pathways to teenage childbearing. Some cases of teenage childbearing may indeed reflect lack of contraceptive education; our results merely show that this is not generally the case in this cohort. Moreover, we have not discriminated the possibility that, for example, one subset of teenage conceptions is preceded by depression in childhood, while a different subset is preceded by early menarche, from the possibility that depression in childhood causes early menarche which leads to early parenthood. Our data are also relatively old, with the NCDS young mothers having their babies in the 1970s. Although the UK rate of teenage childbearing has declined since that time [28], there is no reason to believe that fundamental socioeconomic or psychosocial determinants have altered significantly in recent decades [62]. Indeed, one influential study of teenaged mothers in contemporary Britain noted that they continue to experience difficulties similar to those reported for earlier cohorts. Moffitt and E-Risk Study Team [63] reported that mothers who gave birth at or before age 20 were more socioeconomically deprived, had reduced human and social capital and experienced significantly more mental health problems than mothers who delayed childbearing.

The current research is valuable for two reasons. First, it allows us to clearly identify individual-level developmental precursors of early childbearing, above and beyond socioeconomic background. Our results suggest that young women who physically mature earlier in comparison to their peers, and especially those whose emotional and behavioural adjustment before puberty is poor, are at substantially increased likelihood of seeking early parenthood. Second, it has implications for the design of interventions. One of the few respects in which the future young mothers did not, on aggregate, differ significantly from the controls is in their exposure to sex education lessons about conception, or their satisfaction with those lessons (cf. [1]). Moreover, the finding that future young mothers had earlier ideal ages for parenthood undermines the view that teenage pregnancy is generally caused by mistakes stemming from poor contraceptive skills. Instead, teenage childbearing generally occurs in the context of early target ages for conception, and stands at the culmination of a long developmental trajectory that begins as early as *in utero*. It is quite plausible that interventions that improve birthweight or early growth, or reduce emotional distress in childhood, would disrupt this developmental trajectory, and have the eventual effect of reducing teenage pregnancy rates, while merely improving knowledge of contraception is unlikely to have much effect. This suggestion is borne out by the literature on the effectiveness of different kinds of intervention programme, which shows that interventions aimed at increasing childhood well-being do tend to have an impact [55], whereas sex education programmes aimed at adolescents do not [10–12].
